# Ocean currents influence the genetic structure of an intertidal mollusc in southeastern Australia – implications for predicting the movement of passive dispersers across a marine biogeographic barrier

**DOI:** 10.1002/ece3.535

**Published:** 2013-03-25

**Authors:** Adam D Miller, Vincent L Versace, Ty G Matthews, Steven Montgomery, Kate C Bowie

**Affiliations:** 1 Department of Zoology, The University of MelbourneParkville, Melbourne, Victoria, 3010, Australia; 2 School of Life and Environmental Sciences, Deakin UniversityWarrnambool, Victoria, 3280, Australia; 3 School of Information Systems, Deakin UniversityWarrnambool, Victoria, 3280, Australia; 4 Greater Green Triangle University Department of Rural Health, Flinders and Deakin UniversitiesWarrnambool, Victoria, 3280, Australia; 5 Department of Primary Industries NSW, Cronulla Fisheries Research Centre of ExcellencePO Box 21, Cronulla, NSW, 2230, Australia; 6 School of Biological, Earth and Environmental Sciences, University of New South WalesSydney, NSW, 2052, Australia

**Keywords:** Biogeography, *Donax deltoides*, gene flow, life history, microsatellites, mitochondrial DNA, population structure, southeastern Australia

## Abstract

Major disjunctions among marine communities in southeastern Australia have been well documented, although explanations for biogeographic structuring remain uncertain. Converging ocean currents, environmental gradients, and habitat discontinuities have been hypothesized as likely drivers of structuring in many species, although the extent to which species are affected appears largely dependent on specific life histories and ecologies. Understanding these relationships is critical to the management of native and invasive species, and the preservation of evolutionary processes that shape biodiversity in this region. In this study we test the direct influence of ocean currents on the genetic structure of a passive disperser across a major biogeographic barrier. *Donax deltoides* (Veneroida: Donacidae) is an intertidal, soft-sediment mollusc and an ideal surrogate for testing this relationship, given its lack of habitat constraints in this region, and its immense dispersal potential driven by year-long spawning and long-lived planktonic larvae. We assessed allele frequencies at 10 polymorphic microsatellite loci across 11 sample locations spanning the barrier region and identified genetic structure consistent with the major ocean currents of southeastern Australia. Analysis of mitochondrial DNA sequence data indicated no evidence of genetic structuring, but signatures of a species range expansion corresponding with historical inundations of the Bassian Isthmus. Our results indicate that ocean currents are likely to be the most influential factor affecting the genetic structure of *D. deltoides* and a likely physical barrier for passive dispersing marine fauna generally in southeastern Australia.

## Introduction

In many regions of the world discrete faunal boundaries have been identified in marine communities, including the Isthmus of Panama (Knowlton and Weigt 1998; Lessios 2008), the Sunda and Sahul shelves (Lohman et al. 2011), and the Benguela upwelling (Gibbons et al. 2010). Ocean currents, thermal gradients, habitat discontinuities, and paleo-land connections are recognized as some of the potential physical factors driving biogeographic structuring in these regions. However, the extent to which species ranges and gene flow are influenced by these factors is generally limited by species life history traits and ecology (Avise 1996; Sivasundar and Palumbi 2010; Luiz et al. 2012). Understanding the link between biological and physical factors that limit species dispersal and colonization is critical for natural resource management. This provides a framework for managing threatened, invasive, and commercially important species, and insight into how ecological communities are likely to respond to environmental disturbances (e.g., climate change).

**Figure 1:**
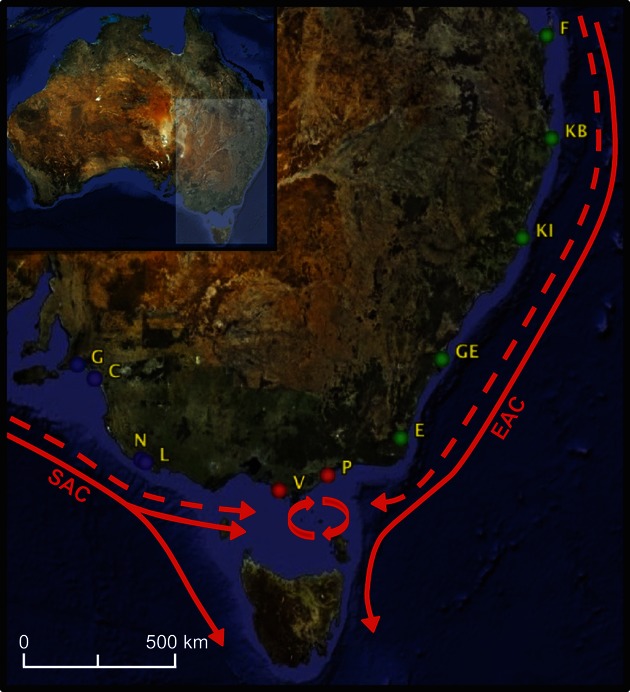
Map of *Donax deltoides* collection sites from south-eastern Australia. G, Goolwa; C, Coorong; N, Nelson; L, Lake Monibeong; V, Venus Bay; P, Paradise Beach; E, Eden; G, Gerroa; KI, Kingscliff; KB, Killick Beach; F, Fraser Island. Color coding of collection sites correspond with genetic clusters identified by Bayesian analyses (blue, western population; green, eastern population; red, admixed genotypes). Solid red lines represent the direction of dominant ocean currents (SAC = South Australian Current; EAC = East Australian Current), dashed red lines represent the direction of genotype influx into Bass Strait, and rotating red arrows represent the contact zone between the isolated western and eastern populations.

Major marine biogeographic divides have been well documented in southern Australia (Whitley 1932; Bennett and Pope 1953; Lyne et al. 1996), one of which is known to occur in the Wilsons Promontory region of eastern Victoria. Community assemblages either side of barrier region have been shown to vary significantly and genetic studies have demonstrated that gene flow between conspecifics spanning the barrier is often limited (Ayre et al. 2009; Colton and Swearer 2012). The causes of faunal disjunctions in this region are not certain and are likely to be a result of a complex of historical and contemporary physical factors. Wilsons Promontory is the historical base of the Pleistocene land bridge, the Bassian Isthmus, which historically connected Tasmania with mainland Australia (Lambeck and Chappell 2001). The land bridge would have created a barrier to east-west gene flow, and has been attributed to vicariant divergence and allopatric speciation in some taxa (Waters and Roy 2003; Waters 2008; York et al. 2008). Oceanographic and habitat discontinuities have also been hypothesized as potential drivers of phylogeographic structuring in the region. Here, three major ocean currents converge, the East Australian Current (EAC), the South Australian Current (SAC); and sub-Antarctic surface water (SASW); creating significant environmental gradients (temperature and salinity), and a complex of currents and eddies in Bass Strait waters that separate Tasmania from the mainland (Baines et al. 1983; Ridgway and Godfrey 1997; Ridgway and Condie 2004; Sandery and Kämpf 2007; Fig. 1). This area is also characterized by large stretches of discontinuous intertidal reef habitat separated by large expanses of high-energy sandy beaches. These sand beach habitats extend east of Wilsons Promontory to Ninety Mile Beach and are considered to be a physical barrier to gene flow for many intertidal species, particularly rocky-reef specialists (York et al. 2008; Ayre et al. 2009).

Historical structuring across the Bassian divide has been investigated for a number of marine species using mitochondrial DNA (mtDNA) markers. These markers have revealed deep phylogenetic splits in molluscs, echinoderms, arthropods, and cnidarians, indicating historical vicariant divergence likely associated with the Bassian Isthmus (Waters and Roy 2003; Dawson 2005; Waters et al. 2005; York et al. 2008). However, studies of contemporary structuring using allozyme and microsatellite data indicate that despite opportunities for secondary contact following the inundation of the Isthmus, east-west dispersal remains limited for many species (Billingham and Ayre 1996; Colgan and Paxton 1997; Huang et al. 2000; Kassahn et al. 2003; York et al. 2008). Evidence from the literature suggests that the ability of species to disperse across the Bassian divide is largely dependent on species life histories. Generally, dispersal limitations appear greater in passive dispersers, such as species with planktonic larvae, compared with species with more mobile adult forms (Ward and Elliot 2001; Meggs et al. 2003; Ayre et al. 2009; Shaddick et al. 2011). However, a direct relationship between life history and dispersal does not always apply, as habitat availability also appears to be a key determining factor of gene flow in this region (Ayre et al. 2009). Intertidal rocky-reef invertebrates with relaxed habitat specificity can potentially disperse across the barrier regardless of life history (i.e., direct developer or planktonic larvae). Conversely, gene flow appears limited in intertidal reef specialists with strict habitat requirements (Ayre et al. 2009). While these studies provide valuable insight into the link between biological and physical factors that determine species dispersal in the region there is a significant bias in the literature toward studies of invertebrate rocky-reef specialists. Further genetic investigations involving taxa from pelagic and soft-sediment habitats will help consolidate the generality of patterns.

*Donax deltoides* Lamarck, 1818, is a commercially important bivalve mollusc that is common to high-energy sand beaches in southeastern Australia. Its coastal distribution on mainland Australia extends from southern Queensland more than 3000 km south along the coast to the mouth of the Murray River in South Australia (Murray-Jones and Ayre 1997). Populations are also known to persist on the northern and eastern coasts of Tasmania, and Flinders and King Islands in Bass Strait (Grove 2011). *Donax deltoides* is dioecious, primarily out-crossing, and reproduces sexually by external fertilization. Although information on species fecundity is limited, it is likely to be high due to continuous spawning that occurs throughout the year (King 1995). Adult movement facilitated by wave activity has been documented (Ferguson and Mayfield 2006; T. G. Matthews pers. comm.), however, a planktonic larval phase lasting 6–8 weeks is recognized as the primary mode of dispersal (King 1975). Previous genetic studies indicate that gene flow among *D. deltoides* populations is likely to be extensive over large geographic distances and directly influenced by ocean currents. Analyses of populations separated by up to 1500 km along the New South Wales coastline indicate panmixia driven by ocean currents associated with the EAC on the east coast of Australia (Murray-Jones and Ayre 1997).

Gene flow and population structuring of *D. deltoides* between New South Wales and South Australia has not been previously investigated. While the conflicting ocean currents of Bass Strait pose a potential barrier to gene flow, the influence of habitat on genetic structuring is likely to be minimal. Unlike previous genetic studies of intertidal reef-dependent invertebrates that have sporadic distributions in the region due to limited habitat availability, large populations of *D. deltoides* are found on sandy beach habitats either side of Wilsons Promontory, including Ninety Mile Beach. This unrestricted habitat availability, coupled with the generation of long-lived planktonic larvae year round, suggests that *D. deltoides* is an ideal model species for testing the influence of ocean currents on the structuring of passively dispersing marine fauna in southeastern Australia.

The present study describes broad patterns of genetic structure of *D. deltoides* from 11 locations spanning the Bassian divide in southeastern Australia. Patterns of contemporary and historical structuring were assessed using 10 polymorphic microsatellite loci and mitochondrial DNA sequence data, and contrasted to gain insight into the demographic history of the species. We test the hypothesis that major ocean currents in southeastern Australia (EAC and SAC) directly influence contemporary structuring of *D. deltoides,* and compare these findings with those from species with various life histories to identify likely common drivers of gene flow in the region. We discuss these findings in context of biogeography, biodiversity conservation, and fisheries management of *D. deltoides* Australia.

## Materials and Methods

### Sample collection and DNA extraction

*Donax deltoides* specimens were collected from 11 locations extending from Fraser Island in Queensland to Goolwa in South Australia (Table 1). Approximately 40 specimens were collected by hand along a 400 m transect from each site. To ensure true population representation and avoid sampling, single cohort specimens representing various size classes were collected. Samples were transported to the laboratory on ice and subsequently stored at −20°C.

**Table 1:** Collection details for *Donax deltoides* samples included in this study. Number of individuals for which microsatellite and mitochondrial genotypes were obtained (*n*), and the haplotype frequencies are also provided

Population	State	Code	Longitude	Latitude	n (Msat)	n (mtDNA)	mtDNA haplotype frequencies
Goolwa	South Australia	G	−35.525	138.78	30	10	1(0.30), 2(0.40), 13(0.20), 23(0.10)
Coorong	South Australia	C	−35.91	139.395	35	10	1(0.70), 13(0.30)
Nelson	Victoria	N	−38.067	141.014	24	12	1(0.58), 2(0.08), 3(0.08), 10(0.08), 15(0.08), 23(0.08)
Lake Monibeong	Victoria	L	−38.141	141.176	26	9	1(0.44), 2(0.11), 13(0.22), 14(0.11), 20(0.11)
Venus Bay	Victoria	V	−38.706	145.811	29	13	1(0.69), 2(0.08), 4(1.08), 6(0.08), 10(0.08)
Paradise Beach	Victoria	P	−38.195	147.423	29	10	1(0.80), 2(0.10), 6(0.10)
Eden	New South Wales	E	−37.006	149.758	14	10	1(0.20), 2(0.20), 5(0.10), 6(0.10), 8(0.10), 16(0.10), 21(0.10), 22(0.10), 25(0.10)
Gerroa	New South Wales	GE	−34.773	150.813	30	10	1(0.10), 2(0.30), 5(0.10), 7(0.10), 8(0.20), 18(0.10), 26(0.10)
Kingscliff	New South Wales	KI	−31.187	152.978	30	8	1(0.25), 5(0.13), 8(0.25), 11(0.13), 26(0.13), 27(0.13)
Killick Beach	New South Wales	KB	−28.254	153.576	30	11	1(0.18), 8(0.27), 12(0.09), 17(0.18), 18(0.09), 20(0.09), 26(0.09)
Fraser Island	Queensland	F	−25.22	153.13	30	8	1(0.25), 2(0.13), 8(0.38), 9(0.13), 19(0.13)

Total genomic DNA was extracted from 30 individuals per population using a modified Chelex® extraction protocol (Walsh et al. 1991). Using a 0.5 mL Eppendorf tube, ∼10 mg tissue sample was taken from the foot, macerated with a scalpel, combined with 150 μL of 5% Chelex (Roche, Melbourne, Australia) solution and 3 μL Proteinase K (10 mg/mL) and mixed briefly by vortex. Samples were incubated at 56°C for 2 h with periodic vortexing, followed by further digestion at 95°C for 15 min. Tissue extractions were cooled on ice for 20 min and stored at −20°C until required for analysis. Prior to polymerase chain reaction (PCR), chelex extractions were homogenized by inversion and centrifuged at 13,000 rpm for 2 min. Supernatant was subsequently taken for PCR from the bottom half of the supernatant above the chelex resin precipitate.

### Microsatellite analysis

Frequency variation of nuclear microsatellite alleles among *D. deltoides* populations was tested to assess gene flow and population genetic structuring. Microsatellite analyses were conducted using 10 polymorphic loci previously developed by Miller et al. (2012). Microsatellite loci were co-amplified by multiplex PCR following procedures described by Blacket et al. (2012).

Descriptive statistics were calculated for the microsatellite data using FSTAT, version 2.9.3 (Goudet 1995) including allelic richness per population averaged over loci, Weir and Cockerham's measure of *F*_IS_, a global estimate of *F*_ST_ (with 95% confidence limits) (Weir and Cockerham 1984), population pairwise measures of *F*_ST_ and their significance determined using permutations (10,000), and pairs of loci tested for linkage disequilibrium using a log-likelihood ratio test. In order to overcome potential limitations of *F*_ST_ calculations using multiallelic loci (Jost 2008) additional estimates of population differentiation, *D*_est_, were obtained using SMOGD version 1.2.2 (Crawford 2010). The software MICRO-CHECKER (Van Oosterhout et al. 2004) was used to assess microsatellite loci for null alleles and scoring errors using the formulas outlined by Brookfield (1996). The sequential Bonferroni procedure (Rice 1989) was used to adjust significance levels when performing multiple simultaneous comparisons.

Estimates of observed (*H*_O_) and expected (*H*_E_) heterozygosity were determined using the Excel Microsatellite Toolkit (Park, 2001) and deviations from Hardy–Weinberg equilibrium (HWE) were tested using Genepop version 3.4 (Raymond and Rousset 1995). An analysis of molecular variation (AMOVA) was performed in GenAlEx (Peakall and Smouse 2006) by using pairwise *F*_ST_ as the distance measure, with 10,000 permutations and missing data for loci set at 10%. The model for analysis partitioned variation among regions (discrete populations identified by microsatellite analysis), among sample sites within regions, and within sample sites. A factorial correspondence analysis (FCA), implemented in GENETIX version 4.05 (Belkhir et al. 2004), was used to summarize patterns of genetic differentiation between sample sites. The first two underlying factors that explain the majority of variation in multi locus genotypes across loci were plotted.

Bayesian analyses were conducted to estimate the number of populations within the sample data using two software packages. First STRUCTURE (Pritchard et al. 2000) was used to identify the number of distinct clusters/populations, assign individuals to clusters, and to identify migrants and admixed individuals using genetic data only. To determine the number of populations (*K*) ten independent simulations for *K* = 1–11 with 100,000 burn-in and 1,000,000 data iterations were run. Analyses were performed using the admixture model of population structure (i.e., each individual draws some fraction of their genome from each of the *K* populations) and allele frequencies were set as independent among populations. The most likely *K* was estimated using Evanno's Δ*K* (Evanno et al. 2005). Bayesian analysis of population genetic structure was also performed using the R-package software TESS (Durand et al. 2009). This method makes use of a geographically constrained Bayesian model that explicitly takes into account the spatial position of sampled multilocus genotypes without any prior information on the number of populations and degree of differentiation between them. Like STRUCTURE, *K* can be treated as variable enabling the determination of the modal (i.e., most likely) value. A pilot analysis was performed initially to confirm that 50,000 sweeps with a 10,000 step burn-in stabilized the likelihood. *K* was then determined from five independent runs where the value was allowed to vary from 1 to11. After identifying the most likely *K*, 100 replicate analyses were performed using an admixture model and summarized using CLUMPP (Jakobsson and Rosenberg 2007).

Evidence of population bottlenecks was investigated using the software BOTTLENECK 1.2.02 (Cornuet and Luikart 1997). BOTTLENECK tests for the departure from mutation-drift equilibrium based on heterozygosity excess or deficiency. In a population at mutation-drift equilibrium there is approximately an equal probability that a locus shows a gene diversity excess or a gene diversity deficit.

This analysis does not require a priori information on historical population sizes or levels of genetic variation. It requires only measurement of allele frequencies from 5 to 20 polymorphic loci in a sample of approximately 20–30 individuals. The analysis compares heterozygosity expected (*H*_E_) at HWE to the heterozygosity expected (*H*_EQ_) at mutation-drift equilibrium for each sample locality. The significance of any observed excess was tested using a Wilcoxon's sign-rank test based on the stepwise mutation (SMM) and two-phase mutation (TPM) models (Di Rienzo et al. 1994) following 1000 iterations.

### Mitochondrial DNA analysis

Approximately 650 base pairs (bp) of the mitochondrial cytochrome oxidase I gene (*COI*) was amplified for 10 individuals from each sample site by PCR using primers HCO and LCO (Folmer et al. 1994). PCRs were prepared in 25 μL volumes each containing 10.25 μL ddH2O, 1× reaction buffer, 5.0 μL dNTPs (1 mmol/L), 0.4 μmol/L HCO primer (10 μmol/L), 0.4 μmol/L LCO primer (10 μmol/L), 0.25 units NEB taq (Biosciences, New England), and 5 μL DNA extract. PCRs were conducted using an Eppendorf Gradient *S* Master Cycler with cycling conditions consisting of an initial denaturing at 94°C for 2 min, 35 cycles of 94°C for 30 sec, 48°C for 30 sec; 72°C for 50 sec, and a final extension step of 72°C for 5 min. PCR products were directly sequenced in both directions using the primers described above and an ABI 3730 capillary DNA analyzer.

Forward and reverse sequences were aligned and manually edited in Sequencher version 4.6 (Genecodes). Consensus sequences were imported into MEGA version 5.1 (Tamura et al. 2011) for multiple alignments with Clustal W (Larkin et al. 2007) using the default parameters (opening gap penalty = 15 and gap extension penalty = 6.66). Genealogical relationships between mitochondrial haplotypes were inferred from a haplotype network (Templeton et al. 1992). Unrooted networks were generated with TCS version 1.21 (Clement et al. 2000), using maximum parsimony to connect haplotypes with a 95% confidence interval. Arlequin version 3.5 (Excoffier et al. 2005) was used to estimate global *Φ*_ST_ (analogous to *F*_ST_) and pairwise measures of *Φ*_ST_ among sample locations. Significance was determined using 10,000 permutations and significance levels were corrected using the sequential Bonferroni procedure (Rice 1989). An AMOVA was also performed in Arlequin using *Φ*_ST_ as the distance measure and 10,000 permutations. The model for analysis partitioned variation among and within sample locations.

Mismatch distributions were examined to test for signals of demographic expansion in *D. deltoides* populations. Mismatch analysis compares the distribution of pairwise differences between sequences to those expected under Rogers and Harpending's (1992) model of demographic expansion. Populations that have undergone expansion events exhibit unimodal distributions whereas populations at equilibrium tend to exhibit multimodal distributions. We used Arlequin version 3.5 (Excoffier et al. 2005) to generate estimates of tau (τ) and two test statistics for the model of expansion: the raggedness index and sum of squared deviations (SSD) tests. When statistical support for expansion was detected, the timing of these events (τ = 2*μt*, where *μ* is the mutation rate per time, and *t* is the estimated time of expansion) was estimated using an mtDNA COI mutation rate of 0.7–1.2% per million years (Marko 2002), and generation time of 1.08 years (King 1975).

An independent measure of demographic history was estimated using a Bayesian Markov-Chain Monte Carlo (MCMC) coalescent approach implemented in BEAST 1.7.3 (Drummond and Rambaut 2007a). The Bayesian skyline plot uses MCMC sampling procedures to estimate a posterior distribution of effective population size through time from a sample of gene sequences, given a previously specified nucleotide substitution model (Drummond et al. 2005). The time dimension of the analyses was calibrated by fixing the mean substitution rate to 1% per million years (clock rate 0.01), calculated as the approximate mean estimate from Marko (2002). The prior on this rate was set to follow a normal distribution allowing for uncertainty around the estimate. Analyses were run using a best-fit model of evolution (HKY + G) identified under Akaike Information Criterion (Akaike 1973) using MrModeltest (Nylander 2004), 100 million MCMC generations sampled every 1000 generations, and launched from a random starting tree. The analysis was repeated in triplicate and log files were examined using Tracer ver. 1.5 (Drummond and Rambaut 2007b) to determine the appropriate burn-in (10% of chain length), to ensure that runs were returning samples from the same distribution, and to ensure that the effective sample sizes for all demographic statistics were greater than 1000. Post burn-in log and tree files from each independent run were combined using LogCombiner ver. 1.7.3 and the trees in the posterior sample were summarized by Bayesian skyline reconstruction using a stepwise skyline variant.

## Results

### Microsatellite analysis

A total of 307 *D. deltoides* specimens representing 11 sampling locations were successfully genotyped at 10 microsatellite loci. A total of 120 alleles were detected, with a mean of 12 alleles per locus over all sites. Allelic richness over all loci ranged between 4.40 and 9.00 (Table 2). Estimates for total number of alleles and allelic richness at these sites were significantly lower (*P* < 0.001) at South Australian and western Victoria locations (mean values 5.14 and 2.62, respectively) compared with estimates from locations in the more easterly Victorian (Venus Bay and Paradise Beach) and New South Wales locations (mean values 8.32 and 3.60, respectively). Expected heterozygosities were moderate to high and ranged from 0.53 to 0.71 (mean *H*_E_ = 0.65), with locations in South Australia and western Victoria also exhibiting the lowest genetic diversity (Table 2). Marker independence was confirmed across all sample sites with linkage disequilibrium analyses indicating no significant linkage between loci (Table 2).

**Table 2:** Statistics for *Donax deltoides* populations screened with nine and five microsatellite loci, respectively (separated by/)

	*a*	*r*	*H*_E_	*H*_O_	HW *P*-value	*F*_IS_	LD *P*-value
G	5.56/5.40	2.59/3.91	0.53/0.59	0.46/0.51	>0.05/>0.05	0.13/0.14	>0.05/>0.05
C	5.56/5.40	2.58/3.89	0.55/0.59	0.51/0.57	>0.05/>0.05	0.08/0.03	>0.05/>0.05
N	5.11/5.60	2.73/4.44	0.58/0.61	0.54/0.55	>0.05/>0.05	0.06/0.10	>0.05/>0.05
L	4.33/4.20	2.59/3.73	0.55/0.57	0.47/0.49	<0.05/>0.05	0.14/0.14	>0.05/>0.05
V	7.67/7.00	3.18/5.20	0.63/0.62	0.60/0.62	>0.05/>0.05	0.04/0.00	>0.05/>0.05
P	8.22/7.20	3.43/5.56	0.67/0.63	0.62/0.60	**<0.001**/>0.05	0.07/0.05	>0.05/>0.05
E	6.22/6.00	3.44/5.51	0.69/0.66	0.53/0.55	**<0.001**/>0.05	0.25/0.19	>0.05/>0.05
GE	9.44/8.40	3.69/5.89	0.73/0.69	0.67/0.67	<0.05/>0.05	0.09/0.04	>0.05/>0.05
KI	9.22/8.20	3.76/5.99	0.75/0.70	0.63/0.69	**<0.001**/>0.05	0.16/0.02	>0.05/>0.05
KB	9.11/7.80	3.82/6.04	0.76/0.72	0.68/0.68	**<0.001**/>0.05	0.10/0.05	>0.05/>0.05
F	8.33/8.40	3.58/5.88	0.71/0.69	0.63/0.64	**<0.001**/>0.05	0.11/0.07	>0.05/>0.05

Mean values over loci are presented for number of alleles (*a*), allelic richness (*r*), observed (*H*_O_) and expected (*H*_E_) heterozygosities, inbreeding (*F*_IS_), Hardy–Weinberg equilibrium *P* values (significance after corrections for multiple comparisons indicated by bold text), and linkage disequilibrium *P* values.

Significant departures from HWE were observed (*P* < 0.05 across all loci) for 8 of the 11 locations, while only five were significant after corrections for multiple comparisons (Table 2). These values were also accompanied by significant *F*_IS_ values, and all locations except for Venus Bay were characterized by a significant excess of homozygotes. These estimates appeared to be largely affected by a small number of loci when HWE estimates at the locus and population level were assessed, with significant departures from HWE at locus DD6 for all sample sites.

MICRO-CHECKER analyses indicated potential influence of null alleles at locus DD6 in all populations. Evidence of potential null alleles in some populations was also observed at loci DD12, DD14, DD15, DD24, although evidence of null homozygotes was not observed. In order to avoid potentially biasing the results, locus DD6 was excluded from further analysis and independent analyses were performed including and excluding the remaining potentially problematic loci. Results from each analysis indicate the exclusion of loci 12, 14, 15, and 24 has no significant affect on the overall results. Therefore, the results discussed below are derived from the analysis including all loci except locus DD6 (statistics for reduced locus analysis are also summarized in Table 2).

Global estimates of *F*_ST_ and *D*_est_ across all loci were significantly different from zero (*F*_ST_ = 0.07; 95% CI = 0.05–0.11; *D*_est_ = 0.19; 95% CI = 0.17–0.29) indicating limited gene flow and genetic structuring amongst sampling locales. Pairwise population comparisons of *F*_ST_ indicate limited gene flow among three distinct regions (western = G, C, N, L; central = V, P; eastern = E, GE, KI, KB, F) with significant values observed at all pairwise comparisons among these regions. Conversely, all pairwise comparisons of sites within regions did not differ significantly from zero indicating panmixia (Table 3). An AMOVA showed significant differentiation between these regions (Western, Central, Eastern), between populations within regions as well as differences within populations. The majority of the variation in microsatellite loci was explained by variation within populations (83%; *P* < 0.001), whereas variation between regions (western, central, and eastern) explained 16% (*P* < 0.001) of the variation, and populations within regions explained 1.0% (*P* < 0.001). The high “between region” variation emphasizes the extent of genetic differentiation among *D. deltoides* populations from different Australian coastal areas.

**Table 3:** Pairwise genetic distances among *Donax deltoides* populations

	C	G	N	L	V	P	E	GE	KI	KB	F
G	*	0.00	0.00	0.05	**0.09**	**0.10**	**0.14**	**0.15**	**0.17**	**0.14**	**0.16**
C	−0.01	*	0.00	0.06	**0.08**	**0.09**	**0.11**	**0.13**	**0.16**	**0.12**	**0.14**
N	0.00	0.00	*	0.03	**0.10**	**0.10**	**0.12**	**0.13**	**0.14**	**0.12**	**0.13**
L	−0.01	0.00	0.00	*	**0.09**	**0.09**	**0.12**	**0.14**	**0.16**	**0.13**	**0.15**
V	**0.09**	**0.10**	**0.11**	**0.07**	*	0.01	**0.05**	**0.04**	**0.06**	**0.04**	**0.05**
P	**0.10**	**0.11**	**0.11**	**0.08**	0.01	*	0.01	**0.02**	**0.03**	0.02	**0.02**
E	**0.12**	**0.14**	**0.14**	**0.10**	**0.06**	0.03	*	0.00	0.00	0.00	0.01
GE	**0.12**	**0.14**	**0.14**	**0.10**	**0.03**	**0.03**	0.01	*	0.00	-0.01	0.00
KI	**0.15**	**0.16**	**0.17**	**0.14**	**0.05**	**0.04**	0.01	0.00	*	0.00	0.00
KB	**0.12**	**0.13**	**0.14**	**0.10**	**0.03**	0.02	0.01	-0.01	0.00	*	0.00
F	**0.15**	**0.17**	**0.17**	**0.14**	**0.05**	**0.03**	0.01	0.00	0.00	0.00	*

Pairwise *F*_ST_ for microsatellite datasets consisting of 9 and 5 loci are given in the lower and upper diagonals, respectively. *F*_ST_ values shown in bold are significant (*P* < 0.001) after 10,000 permutations and corrections for multiple comparisons.

**Figure 2:**
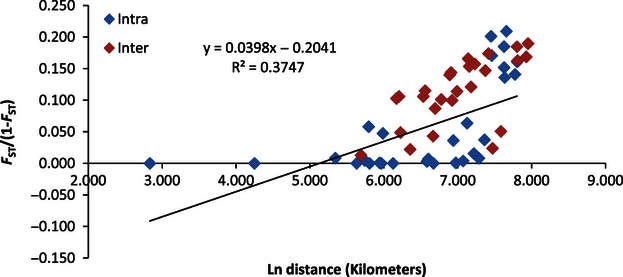
Regression analysis for the *Donax deltoides* microsatellite dataset, linearized *F*_*ST*_ against the natural log of the pairwise geographical distance (km). Different colors differentiate inter and intraregion data points. Scores for the accompanying mantel testis *r* = 0.58, *P* = 0.001. Comparatively, regression analyses and mantel tests restricted to the western and eastern populations with not significant, *R*^2^ = 0.26, *r* = −0.49, *P* = 0.882, and *R*^2^ = 0.58, *r* = 0.76, *P* = 0.13, respectively.

**Figure 3:**
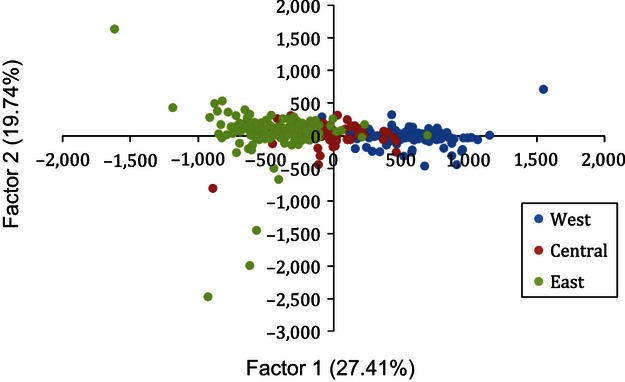
Two-dimensional scatter plot showing the relationships among *Donax deltoides* sample locales based on a factorial correspondence analysis of nine microsatellite loci for 11 sites. The first factor (*x* axis) explains 27.41% of the variance, whilst the second factor (*y* axis) explains 19.74%.

Regression analyses and a Mantel test suggest moderate isolation by distance. There was a significant association between genetic distance and geographic distance with the Mantel test showing a moderate relationship between Slatkin's linearized *F*_ST_ and the natural log of geographic distance (Mantel *r =* 0.70, *P* < 0.01). Regression showed this relationship to be positive and linear (*R*^*2*^ = 0.50; Fig. 2). This signal is lost when analyses are restricted to regions (western- *R*^2^ = 0.26, *r* = −0.49, *P* = 0.882; eastern- *R*^2^ = 0.58, *r* = 0.76, *P* = 0.13) indicating extensive gene flow within regions. The relationships between sample locales are best depicted by the two-dimensional FCA of the microsatellite variation (Fig. 3). When the two factors that explain the majority of the microsatellite variation (factor 1 = 27.41%, factor 2 = 19.73%) are plotted against each other, sample regions separate out along the *x*-axis. There appears to be separation of individuals collected from New South Wales (east) and those from South Australia and Victoria (west) with an area of strong overlap occurring around the Wilsons Promontory region (central). This pattern is somewhat consistent with an isolation-by-distance model, but in this case more likely to be reflecting an isolation-by-barrier scenario.

**Figure 4:**
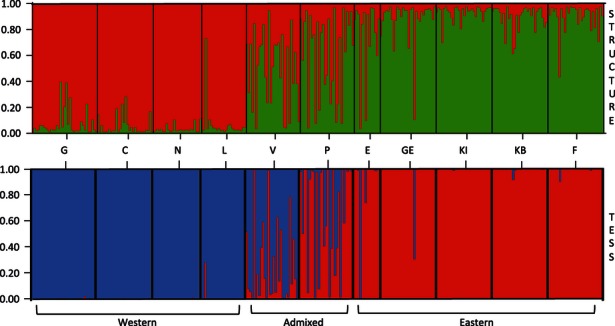
Structure and TESS summary plots of the estimated membership coefficient (*y* axis) for each individual in each two population clusters. Each individual is represented by a single vertical line broken into segments, where segments are proportional to the membership coefficient for each of the population clusters. Individuals are arranged into sites from which they were sampled following the order given in Table 1, and sites are pooled into regions (western, admixed, eastern).

STRUCTURE and TESS Bayesian clustering analyses were congruent, despite the different approaches to identify the number of populations (*K*) within the data. STRUCTURE identified two populations corresponding exactly with those identified by TESS (Fig. 4). These populations appear consistent with pairwise *F*_ST_ estimates and show clear clustering of sites separated by Bass Strait (Table 3). Consistent with FCA analysis, STRUCTURE and TESS outputs also indicate admixture at sites V and P providing strong support for the occurrence of a mixing zone between the eastern and western populations within Bass Strait waters.

BOTTLENECK analysis found no evidence of recent bottlenecks across sample localities. Under each mutation model Wilcoxon's sign-rank test was nonsignificant (*P* = 0.54–0.99 based on SMM and *P* = 0.15–0.99 based on TPM) indicating each site is at mutation-drift equilibrium. These findings indicate that effective population sizes have remained constant in the recent past.

### Mitochondrial DNA analysis

**Figure 5:**
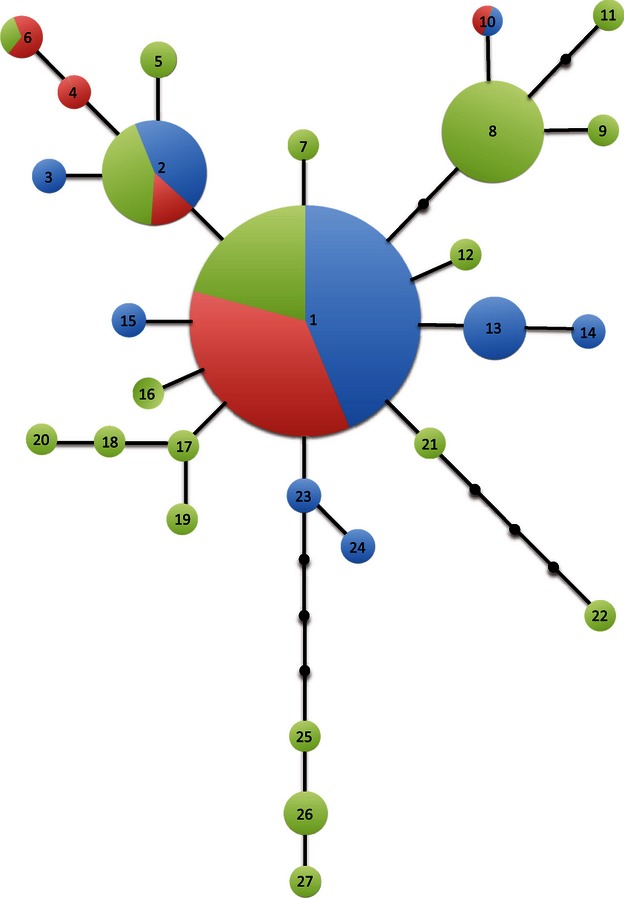
Haplotype network reconstructed by maximum parsimony. Each circle represents a unique haplotype and sizes are roughly proportional to haplotype frequency within the dataset. Black shaded circles on connecting branches between haplotypes indicate single base mutations. Colors indicate region where haplotypes were detected (blue, west; green, east; red, central mixing zone).

A approximately 650 base pair (bp) fragment of the mitochondrial COI gene was successfully amplified and sequenced for 118 individuals representing 11 populations of *D. deltoides* from southeastern Australia (Table 1). Following editing our DNA sequence alignment yielded a 557 bp fragment for analysis. Subsequent genetic analyses revealed 27 haplotypes with a single dominant haplotype (haplotype 1) representing 40% of samples evident and distributed broadly across all sample locations. Unlike the microsatellite analyses the haplotype network revealed minimal genetic differentiation between populations and no geographical pattern associated with the distribution of haplotypes (Fig. 5).

The majority of the variation in the mtDNA dataset was explained by variation within sample sites (94%; *P* < 0.001), whereas variation between sample sites explained only 6% (*P* < 0.001) of the total variation. The low “between site” variation indicates a lack of genetic differentiation among *D. deltoides* collection sites. Global *Φ*_ST_ was weak (*Φ*_ST_ = 0.06) yet significantly different from zero indicating potentially limited gene flow and genetic structuring between sample locations. However, all pairwise comparisons of *Φ*_ST_ were nonsignificant suggesting potential panmixia, or alternatively a lack of statistical power for resolving patterns of genetic structure with confidence.

**Figure 6:**
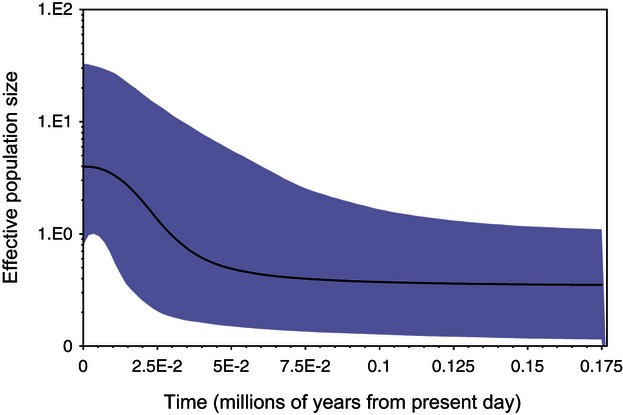
Bayesian skyline plot showing demographic history of *Donax deltoides*. The thick solid line is the median estimate while the shaded area overlay represents the 95% highest posterior density limits.

The demographic history of *D. deltoides* was inferred from mismatch and Bayesian skyline analyses. A model of population expansion was accepted by mismatch analysis, supported by both the raggedness index and SSD tests (*P* = 0.92[*r*]; *P* = 0.69 [SSD]). Using the tau statistic (0.746) the time since expansion was estimated as 51,805–88,736 years. Similarly the Bayesian skyline plot indicates that the *D. deltoides* population had been stable prior to a range expansion event occurring ∼50,000 years ago (Fig. 6).

## Discussion

### Population structure and evolutionary history

Genetic structure of *D. deltoides* across 11 locations was assessed using allele frequencies from nine polymorphic microsatellite loci. A two-step analysis of the microsatellite data was implemented to avoid biases associated with heterozygote deficits at some loci. Heterozygote deficits are common in marine molluscs, particularly bivalve species (Brownlow et al. 2008). The cause of this phenomenon remains uncertain, however, this can be influenced by a range of factors including nonrandom mating, selection, Wahlund effects, and null alleles (Andrade et al. 2005). Heterozygote deficit due to nonrandom mating or Wahlund effects is unlikely in this case as deficits were heterogeneous among loci (significant and nonsignificant *F*_IS_ values). The potential influence of selection is more difficult to determine. Microsatellite loci are typically recognized as neutral genetic markers, however, it is possible that one or more loci are linked to genes or gene groups under selection (Astanei et al. 2005). As has been reported for many other bivalve species, our analyses indicate that a number of loci are potentially influenced by null alleles. These are thought to be common in bivalve species and possibly associated with high mutation rates (Bierne et al. 2003). Although this is a plausible explanation there was no evidence of null homozygotes across loci and duplicate genotypes or direct sequencing across priming sites would be needed to provide confirmation.

Both microsatellite analyses were highly congruent providing confidence in the patterns of genetic structuring that have emerged for *D. deltoides* in southeastern Australia. Each analysis was consistent in identifying two isolated populations and a clear mixing zone. Evidence of a single panmictic population stretching almost 1500 km from Fraser Island (Queensland) along the east coast of Australia to Eden (southern New South Wales) suggests extensive gene flow along the east coast of Australia, further complementing the previous findings of Murray-Jones and Ayre (1997). Sites from the south coast of Australia, including those from South Australia and western Victoria, constitute a second isolated population. This population is characterized by significantly lower estimates of genetic diversity (heterozygosity, number of alleles, allelic richness, number of mitochondrial haplotypes) compared with the east coast population. Bayesian and FCA analyses provide clear evidence of a mixing zone between the eastern and western populations at Venus Bay (Wilsons Promontory region) and Paradise Beach (Ninety Mile Beach). Although, pairwise *F*_ST_ estimates showed weak yet significant support for the isolation of the Venus Bay and Paradise Beach sites, these estimates are likely to be an artifact of mixed ancestral genotypes and should be treated cautiously. Isolation-by-distance modeling was found to be nonsignificant within the eastern and western populations suggesting extensive gene flow over large distances within populations.

The patterns of gene flow and population genetic structure revealed in this study are consistent with the trajectory of major ocean currents in southeastern Australia and the notion that planktonic larval movement is the primary dispersal life stage of *D. deltoides*. Our results indicate that the EAC and SAC currents are key drivers of gene flow on the east and south coasts of Australia, respectively. Gene flow appears extensive and sufficient enough to homogenize microsatellite frequencies over large geographic distances (>1500 km). The direction of larval movement is likely to be largely determined by the predominant current directions (EAC- southward, SAC- eastward), however, bidirectional gene flow is likely within the respective populations given the nonsignificant results from our isolation-by-distance tests. Given the longevity of the larval phase and the year-long spawning cycle of *D. deltoides* it is possible the direction of larval movement is influenced by seasonal variation in current intensity and direction, surface currents driven by varying wind activities, and smaller scale eddie systems (Tilburg et al. 2001). Migration against the predominant ocean currents in southeastern Australia has been previously reported for the barnacle, *Catomerus polymerus*, based on migration patterns inferred from microsatellite data (York et al. 2008).

Evidence of admixture between populations separated by the Bassian biogeographic divide is a particularly interesting finding. Previous studies on intertidal barnacles and snails have suggested possible mixing of populations within the region (Spencer et al. 2007; York et al. 2008), however, an example of definite admixture has not been previously described. As *D. deltoides* dispersal appears largely limited by ocean currents, it is expected that the western and eastern populations should come into contact given the confluence of ocean currents within Bass Strait. For at least 6 months of the year the SAC moves water eastward from the Great Australian Bight into western Bass Strait (Sandery and Kämpf 2007), while periodic intrusions of the EAC into north-eastern Bass Strait are likely to occur (Newell 1960), although this is not well documented. This study provides further support for EAC intrusions, at least periodically allowing east coast *D. deltoides* genotypes to enter Bass Strait waters. While additional sampling would be needed to determine the true extent of the mixing zone, the present study indicates that admixed individuals and their offspring are likely to remain confined to the turbulent waters of Bass Strait, while the ancestral populations remain isolated by contemporary physical conditions. Also given HWE and LD estimates at admixed locations were found to be nonsignificant we expect that recruits from the eastern and western populations are interbreeding freely. Significant values would be expected due to Wahlund effects if recruits from the divergent gene pools were reproductively isolated.

Unlike the microsatellite dataset the mtDNA sequence analysis did not identify patterns of genetic differentiation among the 11 sample locations included in this study. These findings likely indicate historical gene flow and a shared recent common ancestor. Given sex-biased dispersal is extremely unlikely in this species the conflicting patterns of genetic structure derived from mtDNA and microsatellite markers is likely attributed to marker sensitivity. Mitochondrial markers accumulate mutations at much slower rates compared with microsatellite loci (Avise 2004) enabling historical and contemporary patterns of population structure to be contrasted. Consequently, this provides valuable insight into the evolutionary history of the species. Our findings suggest that the contemporary population subdivisions found using microsatellites have established within the last 90,000 years, a period that appears too recent for significant differentiation at the mitochondrial level to accumulate (Lambeck and Chappell 2001).

Estimates of genetic diversity are comparatively lower in the western population at the microsatellite and mitochondrial loci likely reflecting past demographic processes such as bottleneck or founder events. Our analyses did not detect any genetic signature of a historical bottleneck, however, mismatch distribution and Bayesian skyline analyses did provide evidence of a significant range expansion event occurring between 50,000 and 90,000 years ago. Historical sea level models suggest that multiple inundations of the Bassian landbridge occurred within this time period (http://sahultime.monash.edu.au), providing opportunities for expansion events to occur. We propose the western population was likely colonized most recently, involving a relatively small number of founder individuals from the larger, more genetically diverse eastern population. Following this range expansion the western population has remained isolated due to contemporary physical oceanographic conditions. Although highly speculative potential vectors for historical movement across Bass Strait might include irregular ocean currents and climatic events, birds, and even Indigenous Australians who have a long history of harvesting the species as a food resource (Godfrey 1989).

### Physical factors influencing genetic structure

Our findings are consistent with previous studies that suggest contemporary physical conditions of Bass Strait that present a continuing barrier to dispersal for many marine species. Although the bulk of the evidence comes from relatively sedentary invertebrate species (Huang et al. 2000; York et al. 2008; Ayre et al. 2009), contemporary structuring has also been observed in highly mobile species such as fish (Colgan and Paxton 1997), cuttlefish (Kassahn et al. 2003), and jellyfish (Dawson 2005). Strong environmental gradients reported for Bass Strait waters are likely to influence dispersal in some mobile species and play a role in the genetic structuring of marine communities (Colton and Swearer 2012). Consequently, studies of passive dispersers such as those with planktonic larvae provide an independent measure of dispersal limitations associated with trajectory of ocean currents. This is particularly important for predicting the spread of invasive species with planktonic phases, disease, and understanding patterns of dispersal and recruitment in commercially important species such as abalone and rock lobster.

A comprehensive summary of the genetic literature and relationships between life history, ecology, and dispersal in marine species in southeastern Australia is provided by Ayre et al. (2009). Ayre et al. hypothesize that habitat availability rather than larval type is a key determinant of gene flow across the Bassian divide, based on gene flow estimates from 17 intertidal invertebrate species. A total of 11 planktonic dispersers exhibiting strong genetic structuring have been described as rocky-reef specialists (Edgar 2000), for which discontinuous reef habitat stretching 300 km along the coastline is recognized as a likely barrier to gene flow. Conversely, six invertebrate species with available habitat spanning the biogeographic divide showed evidence of gene flow across the Bassian divide (Waters et al. 2007; Ayre et al. 2009). However, the examples described by Ayre et al., are based on gene flow estimates derived from mtDNA data only. Our results indicate that findings based solely on mtDNA data need to be interpreted with some caution, and further studies are needed to strengthen hypotheses. Our study demonstrates the importance of consulting more rapidly evolving markers (e.g., microsatellites, SNPs) to determine contemporary patterns of genetic structuring with greater confidence. Based on mtDNA data alone we could have been misled to believe that *D. deltoides* is a single panmictic unit and capable of dispersing across the biogeographic divide in southeastern Australia. To our knowledge all comprehensive population genetic surveys of planktonic dispersing invertebrates, and species with passively dispersing adult forms, using sensitive genetic markers (microsatellites and allozymes) have identified limited east-west gene flow and significant structuring in this region (Brown 1991; Billingham and Ayre 1996; Huang et al. 2000; Dawson 2005; Sherman et al. 2008; York et al. 2008).

*Donax deltoides* possesses several life history traits that make it an ideal model species for habitat-independent assessments of ocean currents influences on passively moving marine species. Characterized by massive population sizes, continual year-long spawning, long-lived larvae, and extensive habitat availability in southeastern Victoria spanning the biogeographic divide, this species is a prime candidate for negotiating the converging currents and homogenizing gene pools across the divide. Our results indicate that while ecological factors such as habitat are likely to play a key role in the success of colonization in some planktonic dispersers, ocean currents are likely to be major limiting factor of dispersal and driver of contemporary structuring. Despite opportunities for mixing in southeastern Victoria the movement of planktonic larvae across Bass Strait is unlikely regardless of habitat availability.

### Implications for fisheries management

Commercial and recreational fisheries for *D. deltoides* operate across most of its range, and major population declines have been observed in many regions as a result of over-fishing and disease (Murray-Jones 1998; Ferguson and Mayfield 2006; Lewis and Scarpaci 2010). Consequently, this has prompted fishing policy reforms and investments in stock assessment research by respective Australian state governments and the Australian Fisheries Research and Development Corporation (Murray-Jones 1998; Ferguson and Mayfield 2006). Continuing stock declines and increasing fishing pressure highlight the vulnerability of *D. deltoides* and the need for an improved understanding of the biology, ecology, and genetics of this species. In particular, spatial patterns of dispersal and recruitment are needed to devise spatially effective management strategies.

Estimates of contemporary genetic structure as indicated by the microsatellite data in this study provide a valuable framework for guiding future management of *D. deltoides* fisheries. The findings in the present study provide insight into spatial patterns of dispersal and recruitment across the species distribution and a framework for mitigating the threats of over-fishing and disease. The two isolated populations identified effectively represent closed and self-recruiting entities, and are likely characterized by unique adaptive traits. Therefore, it is important that these populations are considered independently when devising management strategies. The results presented here also provide a valuable resource for assessing the resilience of stocks in the admixture zone. Local stocks within this region have been heavily depleted in recent years as a result of over-fishing (Lewis et al. 2012). Our results demonstrate that replenishment of stocks is likely to be influenced by recruitment from source populations located both east and west of Bass Strait. The results generated in this study also provide valuable baseline data for future population monitoring and a spatial framework for guiding potential stock restoration projects including reseeding and translocation activities (Caddy et al. 2003; Weeks et al. 2011).

## Conclusion

We conclude that the major ocean currents of southeastern Australia influence the contemporary structure of *D. deltoides* by limiting east-west dispersal of planktonic larvae across Bass Strait. The immense passive dispersal potential of this species and lack of apparent ecological constraints suggests that gene flow is likely to be restricted in other passive dispersing marine taxa in this region. Ecological factors such as habitat availability are likely to play a role in the colonization success and genetic structure in some species (e.g., intertidal reef specialist taxa), however, we suggest that ocean currents are likely to be a common barrier to dispersal regardless of other ecological factors. This study contributes to our understanding of the physical drivers responsible for the biogeographic structuring of marine communities in southeastern Australia and the link between species life histories and dispersal across marine biogeographic barriers generally.
